# Multiple Myeloma: Heterogeneous in Every Way

**DOI:** 10.3390/cancers13061285

**Published:** 2021-03-13

**Authors:** Anaïs Schavgoulidze, Titouan Cazaubiel, Aurore Perrot, Hervé Avet-Loiseau, Jill Corre

**Affiliations:** 1 Centre de Recherche en Cancérologie de Toulouse, Institut National de la Santé et de la Recherche, Médicale U1037, 31059 Toulouse, France; schavgoulidze.a@chu-toulouse.fr (A.S.); perrot.aurore@iuct-oncopole.fr (A.P.); AvetLoiseau.Herve@iuct-oncopole.fr (H.A.-L.); 2 Hematology Department, University Hospital, 33600 Bordeaux, France; titouan.cazaubiel@chu-bordeaux.fr; 3 Hematology Department, Institut Universitaire du Cancer de Toulouse-Oncopole, University Hospital, 31059 Toulouse, France; 4 Unit for Genomics in Myeloma, Institut Universitaire du Cancer de Toulouse-Oncopole, University Hospital, 31059 Toulouse, France

**Keywords:** multiple myeloma, prognosis, cytogenetics, epigenetics, microenvironment

## Abstract

**Simple Summary:**

With the development of modern therapies in multiple myeloma, prognosis stratification is becoming an indispensable tool for the choice of treatment between patients. Many factors influence the prognosis in multiple myeloma; scores, mainly based on biochemical parameters and cytogenetics, have been proposed to discriminate patients. However, these scores are not perfect and fail to predict some patients’ outcomes. In this review, we describe current evaluated factors and their limitations. In the second part, we address factors with an impact on treatment escape and prognosis, but which are not available routinely yet.

**Abstract:**

Multiple myeloma (MM) is a hematological malignancy characterized by the accumulation of tumor plasma cells (PCs) in the bone marrow (BM). Despite considerable advances in terms of treatment, patients’ prognosis is still very heterogeneous. Cytogenetics and minimal residual disease both have a major impact on prognosis. However, they do not explain all the heterogeneity seen in the outcomes. Their limitations are the result of the emergence of minor subclones missed at diagnosis, detected by sensible methods such as single-cell analysis, but also the non-exploration in the routine practice of the spatial heterogeneity between different clones according to the focal lesions. Moreover, biochemical parameters and cytogenetics do not reflect the whole complexity of MM. Gene expression is influenced by a tight collaboration between cytogenetic events and epigenetic regulation. The microenvironment also has an important impact on the development and the progression of the disease. Some of these determinants have been described as independent prognostic factors and could be used to more accurately predict patient prognosis and response to treatment.

## 1. Introduction

Multiple myeloma (MM) is a cancer characterized by the accumulation of tumor plasma cells (PCs) in the bone marrow (BM). It is always preceded by an asymptomatic state: monoclonal gammopathy of undetermined significance (MGUS). Historically, the term “multiple” was used because of the numerous bone lesions seen at diagnosis. Nowadays, this term also applies to the wide heterogeneity at the molecular level. In these last two decades, considerable progress in patients’ treatment has been made, starting with high-dose therapy intensification supported by autologous stem cell transplantation (ASCT), followed by the development of proteasome inhibitors and immunomodulatory drugs (IMiDs), and now the rise of immunotherapy (anti-CD38, bispecific antibodies, CAR T-cells). Nevertheless, high-risk patients do not really benefit from these improvements and their prognosis stays poor, with survival below 2 years. These heterogeneous results and the complex genomic landscape of myeloma, between patients but also inside the same tumor, suggest that adapting treatment according to age and comorbidities only is out of date. However, surprisingly, some patients relapse early without any explanation given by the current tools such as the Revised International Staging System. Here, we describe the current state of the art regarding prediction of patient clinical outcome and assessment of response, particularly minimal residual disease. In the second part, we discuss the limits of the current tools that could explain failure cases. We describe new data that may contribute to the heterogeneity of the disease and resistance to treatment, from epigenetics to the microenvironment.

## 2. Patient Prognosis: What We Know

Multiple myeloma (MM) is defined by the accumulation of tumor plasma cells (PCs) initially in the BM but also in extra-medullar sites, which secrete a monoclonal immunoglobulin. This accumulation is responsible for numerous symptoms such as bone lesions, anemia or renal failure.

Unlike chronic myeloid leukemia, MM is a hematological malignancy characterized by a wide molecular heterogeneity, between different patients but also within the same patient, with the presence of subclones that differentially grow and evolve. Two entities (most of the time distinct) have been described: on one side, myelomas with translocations which involve the 14q32 locus and, more specifically, the IGH gene, and, on the other side, myelomas with the hyperdiploid karyotype [1]. These abnormalities are considered “primitive”, beginning with the earliest precursor stages; indeed, 100% of MGUSs harbor one or the other.

Numerous factors act as prognostic factors in MM. They are naturally linked to the patient’s fitness (age, comorbidities), which determines the therapeutic strategy, but it is now well understood that the prognosis is mainly influenced by the cytogenetic/molecular abnormalities (CAs) in PCs [2]. Many scores have been developed to stratify patients; the International Myeloma Working Group (IMWG) recommends using the R-ISS. This score has incorporated into the ISS (based on albumin and β2-microglobulin levels) a novel biochemical criterion, lactate dehydrogenase level, and three high-risk CAs: del(17p), t(4;14) and t(14;16) [3].

### 2.1. Cytogenetic Abnormalities

Del(17p) is observed in 8% of newly diagnosed MM patients (NDMM). TP53 is present in the minimally deleted region; nevertheless, the deletion affects only a fraction of the PCs, as a secondary event. It is considered to be the most adverse CA, even if not associated with a TP53 mutation on the second allele [4]. One of the main factors of variability between studies is the threshold value of PCs deleted for 17p, also called the cancer clonal fraction (CCF), which can range from 1 to 60%. The R-ISS does not mention a threshold to classify patients. However, a European meta-analysis confirmed that del(17p) has a prognostic impact with a CCF threshold strictly above 55% [5]. Approximately 5–8% of NDMM patients harbor a monoallelic mutation of TP53. There are various mutations distributed along the whole gene. Frequently, these are missense mutations targeting the DNA binding-domain, inducing an alteration of the protein function. Nonsense and frameshift mutations are rare in MM. The diversity of TP53 mutations and their low frequency could explain the lack of data to assess their prognostic impact. Finally, a biallelic inactivation occurs in 3% of patients and confers the worst prognosis [6]. It is the result of a deletion, 17p, combined with a TP53 mutation on the other allele (“double-hit” myeloma).

Translocation t(4;14)(p16;q32) is the second translocation involving the IGH locus in terms of frequency and is specific to MM. It results in the juxtaposition of FGFR3 and NSD2 with the IGH enhancers. This fusion increases the expression of NSD2 in all patients, and of FGFR3 in 70% of them. Indeed, a loss of FGFR3 is observed in about one third of the cases. Translocation t(4;14) induces a genomic instability; it is commonly associated with other CAs such as gain(1q) and del(13q). Its prognostic impact is heterogeneous and may depend on many factors, such as its co-occurrence with del(1p32) [7] and the breakpoint in the NSD2 gene. Li et al. showed that patients with the entire NSD2 protein had better outcomes than those with a truncated version [8]. It is important to mention that some subgroups of patients harboring t(4;14) have seen an improvement in their prognosis with the introduction of proteasome inhibitors [9], even if their outcomes are still poorer compared to patients without t(4;14).

Translocation t(14;16)(q32;q23), involving the MAF gene, is part of the R-ISS despite contradictory studies about its prognostic impact [10,11]. Indeed, its independent impact on the prognosis has not been proven yet because of its low frequency (3.5% of patients at diagnosis) and its recurrent association with other CAs known to be pejorative, such as del(17p) or gain(1q) [12,13].

The R-ISS, published in 2015, includes only three CAs mainly for two reasons: first, because the construction of the score required the inclusion of broadly measured parameters in the cohorts used; second, because the aim of the score was to be applicable widely around the world. However, other CAs, more or less prevalent, have shown to be very interesting in prognosis assessment. Gain(1q21) (30% of NDMM patients) and del(1p32) (about 10%) are both independent prognostic factors [14,15,16]. An amplification (≥4 copies) of 1q21 (CKS1B) in an ISS-3 background confers to patients a very poor prognosis [6], while the del(1p32) impact is almost as poor as the del(17p) one. In contrast, hyperdiploid karyotypes are generally considered favorable, but this is less simple than it looks. Trisomies 3 and 5 are favorable, but, on the contrary, trisomy 21 seems to be a factor of poor prognosis [17].

Considering the important impact of CAs, the Intergroupe Francophone du Myélome (IFM) has developed a weighted score based on six CAs: del(17p), t(4;14), gain(1q21), del(1p32), trisomy 21 and trisomy 5 [2], the last one being the only protective one. This score showed better performance in risk assessment than R-ISS. Recently, Kuiper et al. successfully improved the performance of R-ISS in older patients by combining it to SKY92, a score based on the expression of 92 genes [18].

### 2.2. Treatment Response and Minimal Residual Disease (MRD)

Although it cannot be part of the initial prognostic assessment, treatment response is, with CAs, a major prognostic factor in MM. Thanks to the evolution of therapies, more and more patients achieve a complete response (CR) after their first treatment. More sensitive and discriminant criteria were needed to deeply assess the treatment response; with the technological advent, it is now possible to measure the minimal residual disease (MRD) either with next-generation sequencing (NGS) or next-generation flow (NGF) with a sensitivity from 10^−5^ to 10^−6^. For both first line and relapse, it was proven that the response was deeper and the outcome was better (progression-free survival and overall survival) [19]. A lot of studies showed that MRD negativity was a strong predictor of clinical outcome [20,21,22]. A recent study showed that MRD can overcome the initial risk stratification. Despite lower rates of undetectable MRD in high-risk patients, those who achieved it had the same outcomes than standard risk patients with an undetectable MRD [23]. However, for patients with a persistent MRD, high-risk patients had poorer outcomes than standard risk ones.

Another crucial factor is the response duration: it has been recently shown that an early relapse after autologous transplant was still associated with poor survival, even after innovative rescue treatment [24]. This introduces the notion of sustained MRD. In the future, MRD negativity may be a driver for tailored treatment, with a therapeutic intensification for those who do not achieve it. Another goal is to validate MRD as a surrogate marker for clinical trials in order to reduce the length of the studies. The main challenge about MRD is to standardize practices: threshold of positivity, technologies and duration for a sustained response.

Unfortunately, despite advances in the knowledge and understanding of myeloma physiopathology, some patients relapse early or are refractory without any explanation predictable by the current risk assessment. This suggests that underlying mechanisms exist and that a single “photograph” of the risk at diagnosis is not satisfactory. First, risk should be seen as a dynamic concept and not like a parameter stuck in time. As with many cancers, clonal PCs under selection pressure can escape the treatment by selecting fitter subclones and/or acquiring new mutations. Genomic instability seems to be higher for high-risk patients [23]. Hypothetically, we could imagine a therapy adapted based on the risk assessed at the beginning, essentially based on CAs, and during the follow-up, with an adjustment according to the MRD status, to avoid the persistence of these cells that could be responsible for early relapse. Secondly, and this is understandable, there are several levels of heterogeneity which are not taken into account routinely. Some aspects surely have an influence on the prognosis, but this influence is not assessable with the current tools.

## 3. MM Heterogeneity as a Powerful Lever for Treatment Escape

### 3.1. Intratumoral Heterogeneity

MM is a clonal disease with a genomic landscape which differs between patients but also in the same tumor. Mutational analysis confirmed the diversity in MM; whole-exome sequencing highlighted an average number of subclones in the same patient of five [23]. This mean value is probably underestimated because the threshold for the subclones’ detection was at least 10%. This diversity increases the risk of resistance and makes the eradication of all PCs difficult. No unifying mutation has been found in MM, unlike other hematological malignancies such as BRAF V600E in hairy cell leukemia or MYD88 L265P in Waldenström macroglobulinemia. In MM, the most frequently mutated genes are KRAS (25%), NRAS (20%), DIS3, BRAF and FAM46C (about 10–12% each) [25,26]. Numerous other genes are mutated in less than 10% of patients, among them TP53. These mutations are considered to be drivers and responsible for the disease progression. The proof of a prognostic impact was demonstrated only for TP53, unsurprisingly unfavorable, although the prognostic value of a monoallelic mutation (without the 17p deletion) remains unclear [27]. Whether other mutations are prognostic markers or not is still unclear. Some of them are targetable with a specific therapy (notably BRAF V600E and vemurafenib, MAPK mutations and MEK inhibitors). On the contrary, some can confer resistance to some therapeutic classes (CRBN mutation and IMiD resistance). In theory, these insights could help to adapt treatment. In reality, the diversity of mutations inside the same tumor brings the risk of a partial eradication and de facto the emergence of resistance.

In addition, some minor subclones are missed at diagnosis because they are undetectable with the current techniques; this could explain the difference between diagnosis and relapse clones. Single-cell RNA sequencing (scRNA-seq) brought another level of sensitivity. Instead of giving a general map of the molecular alterations in the tumor burden, this technic allows the study of alterations cell by cell and accurately defining the diversity of subclones. This method is relatively recent in MM, but some studies provided promising new elements of comprehension. Single-cell RNA-seq confirmed an intratumor transcriptional and copy number alteration heterogeneity, which can be roughly deduced from the transcriptome [26]. Additionally, scRNA-seq can be theoretically applied to the MRD endpoint and provide information about clonal evolution and resistance to treatment mechanisms [23,28]. For patients with detectable residual disease, driver genes (such as CCND1, NSD2 and FRZB) were often found expressed equivalently before and after treatment. Targeted therapies could be proposed for patients with a positive MRD to eradicate the residual tumor cells, depending on their mutational profile. On the contrary, this analysis has also detected the emergence of new mutations, different between patients, such as LCP1 previously reported as a potential driver in chronic lymphocytic leukemia [29] and whose overexpression was presented as a potential prognostic marker by Shin et al. [30]. Of note, Ledergor et al. also used scRNA-seq to characterize rare tumor cells in patients with asymptomatic stages (seven subjects with MGUS and six with smoldering multiple myeloma) [28]. In the future, this highly sensitive approach could help to define which patients have a greater risk of progression and should benefit from treatment at the earliest possible stage.

### 3.2. Temporal Heterogeneity

Despite better results in patients’ outcomes, myeloma remains incurable. It has been shown that during relapses, the genomic profile of PCs could be different from the one at diagnosis or, on the contrary, stay slightly the same (stable evolution). This is called temporal heterogeneity. Several ways of evolution over time have been described. Some patients harbor the same clones between diagnosis and relapse, but the proportion of each clone differs: this evolution is referred to as differential. Under treatment pressure, PCs can adapt to their microenvironment and acquire new mutations: when a new subclone emerges, the evolution is called linear. The last known way of evolution is branching evolution: emergence of one or several subclones while the predominant clone at diagnosis disappears. Dissecting the evolution and adaptation of PCs in their microenvironment and under treatment pressure is critical because it could help to understand and predict part of the resistance in MM [31]. Treatment itself is a driver of therapeutic resistance in many cancers. Chemotherapy kills the most sensitive cells, but a minority of cells can be resistant, survive and expand in a niche left empty by the initial major clone. A study failed to highlight a specific evolution of PCs between diagnosis and relapse from the same intensive treatment in 43 newly diagnosed patients [32]. The effect of treatment seems to be nonspecific and again linked to the heterogeneity between patients from the very start. Chromosomal instability (CIN), recognized as a hallmark of human cancer, is an actor of genomic heterogeneity at every stage of the disease. During progression, it is, in part, responsible for the development of drug resistance [33]. It includes numerical (copy number alterations, amplifications and deletions of chromosome arms) and structural CIN (translocations, duplications, inversions, deletions). MM is greatly affected by these abnormalities. CIN increases with the progression of the disease, which results in the accumulation of CAs from MGUS to MM but also from NDMM to relapsed myeloma [34]. Some non-specific aberrations showed to be independent prognostic factors. Chromothripsis and chromoplexy, which are massive chromosomal rearrangements, were supposed to be rare events in MM (1.3% of patients for chromothripsis). However, whole-genome sequencing revealed higher rates of events (21% for chromothripsis and 30% for chromoplexy), and these alterations are correlated with adverse prognosis [35,36]. Other CINs such as centrosome amplification are associated with poor prognosis.

### 3.3. Spatial Heterogeneity

MM results in the accumulation of clonal PCs in the BM, leading to bone destruction and focal lesions more or less numerous. The number of focal lesions is a prognostic factor because it correlates with the disease progression [37]. Bone marrow is collected mainly from the posterior iliac crest or from the sternum. This invasive act restricts repeated samplings, and the biological investigations are made on a small part of the tumor burden. However, Rasche et al. elegantly showed that spatial genomic heterogeneity was found in the majority of patients at both chromosomal and mutational levels [37]. For example, del(17p) showed spatial variation in two out of six patients. Progression events are often restricted to focal events, whereas initiating events are uniformly spread. The existence of mutations promoting progression since the diagnosis in unexplored regions could partially explain why we observe unpredictable poor outcomes in some patients. The distribution of high-risk disease may be heterogeneous, and the “snapshot” taken at diagnosis only in one locus surely misses important information for risk assessment. The same applies for the assessment of MRD, which may sometimes not be that accurate if it comes from a single sample. This is why imaging-based MRD (PET-CT) may be a good complement to biological MRD, particularly for purely extramedullary localizations [38,39].

These limitations of BM analysis question the development of other technologies which could be able to recapitulate the exhaustive reality. Liquid biopsies have been developed in solid tumors to detect, in a non-invasive manner, circulating tumor cells (CTCs) or directly circulating tumor DNA (ctDNA). Some studies showed a good correlation of the genetic profile between blood and BM analyses in NDMM or relapsed/refractory patients [40,41]. However, the sensitivity is not deep enough for the detection of ctDNA when the tumor bulk is lower because of the contamination with other cells. Mazzotti et al. found no correlation between ctDNA and BM for MRD by NGS looking only at IGH rearrangements [42]. More importantly, more than two thirds of patients with MRD detected in BM had undetectable ctDNA.

### 3.4. Epigenetic, an Encouraging Line of Research

Epigenetic changes affect gene function without altering the DNA sequence. These phenomena are transmissible during cellular divisions but are reversible. They are involved in numerous functions; it is now widely accepted that epigenetic abnormalities contribute to the development and the progression of cancers.

Methylation of DNA is one of the most described systems in epigenetics. It occurs on cytosine residues, especially in CpG (cytosine-phosphodiester bond-guanine) islands, often enriched in the promoter region of genes. Methylated DNA is transcriptionally silenced. In MM, DNA is globally hypomethylated, except on the promoters of tumor suppressor genes (TSG) where DNA is rather hypermethylated [43]. This TSG hypermethylation increases with the progression of the disease, from MGUS to MM and then plasma cell leukemia (PCL). Kaiser et al. identified specific genes (GPX3, RBP1, SPARC, TGFBI) which were independent poor prognostic factors when they were hypermethylated [44]. The re-expression of these genes increased the response to therapy in vitro. DNA methylation is regulated by DNA methyltransferases (DNMT). DNMT1 is highly expressed in MM cells compared to normal plasma cells. DNMT inhibitors such as decitabine or 5-azacytidine are already available for the treatment of myelodysplastic syndromes or some leukemias. In vitro studies have shown a potential efficacy on PCs. Moreaux et al. built a DNA methylation score to assess the levels of methylation in PCs [45]; they showed that this score could be a tool to identify patients who would gain maximum benefit of a treatment by demethylating agents. Nevertheless, to our knowledge, there are no published data of decitabine efficacy on MM patients and only few clinical studies for 5-azacitidine [46,47].

Gene expression is also controlled by histone proteins. Histones form nucleosomes with DNA. The position and condensation of nucleosomes are influenced by the post-translational modifications of histone tails. The “histone code” includes various modifications including acetylation, methylation and phosphorylation and the consequences are as diverse as the enzymes that regulate it. Histone acetyltransferases (HATs) are responsible for active gene expression, whereas histone deacetylases (HDACs) lead to the condensation of the chromatin that impairs the access to the transcriptional machinery. HDACs are overexpressed in MM. This overexpression, particularly for HDAC1, has been associated with poor prognosis in MM [48]. Panobinostat, a “pan inhibitor” of HDAC, is indicated in combination with bortezomib and dexamethasone for relapsed/refractory patients after at least two previous treatments including bortezomib or an IMiD. In theory, panobinostat maintains acetylated histones to keep TSG switched on. The reality is more complex because its activity is wide. Deacetylases are not specific to histones and target thousands of proteins involved in DNA repair, replication, transcription, cell cycle progression, protein degradation, etc. Plenty of actors interfere with the “histone code”. NSD2, overexpressed in t(4;14), interacts with histone proteins and leads to a global alteration of histone patterns, with, for example, the accumulation of H3K36me2 marks, causing transcriptional activation of oncogenic loci [43]. EZH2, frequently upregulated in MM, causes the trimethylation of H3K27 (H3K27me3), drawing repression of gene expression [49]. Pawlyn et al. demonstrated the independent deleterious effect of EZH2 overexpression on clinical outcomes, making it an interesting target [49].

Noncoding RNAs play a major role in the regulation of gene expression. Even if their belonging to epigenetic mechanisms is controversial, it is important to mention micro-RNAs (miR), which have been extensively studied. Unsurprisingly, the miR signature differs between tumor PCs and normal plasma cells, and this signature is more deregulated in MM than MGUS [43]. miR-21 was the first described in MM, and its expression is regulated by the IL-6/STAT3 pathway with a close interaction with bone marrow stromal cells (BMSCs) in the microenvironment [50]. Its overexpression results in inhibition of apoptosis and resistance to melphalan in vitro, while bortezomib could reduce its expression. A growing interest recently emerged on exosomes. Exosomes are nanovesicles containing proteins and nucleic acids such as miR and secreted by cancer cells, allowing them long-distance communication. The analysis of circulating exosomal miRNAs is a non-invasive method to assess miR expression, and some of them (miR-18a, let-7b), associated with poor outcomes, have improved survival prediction in MM patients [51].

### 3.5. The Influence of the Microenvironment

Malignant PCs evolve in niches formed by the BM. These niches, made of bone marrow stromal cells (BMSCs) (adipocytes, osteoblasts), hematopoietic cells (macrophages, lymphocytes) and extracellular matrix proteins, provide a favorable environment for the growth and maintenance of cancer cells. PCs interact closely and reciprocally with their neighbors, particularly through cytokines and growth factors. These interactions are crucial for disease progression [52]. BMSCs in myeloma have a modified phenotype that contributes to the development and the progression of the disease. For example, there is an increased expression of interleukin 6 (IL-6), also produced by PCs, a cytokine promoting the proliferation of B-cells, their differentiation in plasma cells and the activation of osteoclasts, amongst other roles. Despite a wide distribution of focal lesions, PCs stay mostly localized in the BM. The adhesion of MM cells within the niches reduces their access to chemotherapeutic drugs and increases the risk of resistance. CXCL12 is a cytokine that binds to its receptor CXCR4, expressed on many cells including mature plasma cells. A higher expression of CXCL12 was identified in MM, and it could be one of the mechanisms that increases PCs’ homing [52]. These mechanisms constitute potential new targets for the development of therapies. A phase Ib/II trial of the anti-CXCR4 monoclonal antibody ulocuplumab recently showed an acceptable safety profile and a high response rate in combination with lenalidomide and dexamethasone in patients with relapsed/refractory myeloma [53].

BMSCs promote the vascularization of the tumor, and therefore its development, through the secretion of hypoxia-inducible factors (HIF1) which stimulate neoangiogenesis. “Classical” therapies such as IMiDs target the microenvironment with their antiangiogenic properties. Nevertheless, hypoxia is known to induce CIN which exposes PCs to increased DNA damage, mutagenesis and potentially development of drug resistance and tumor progression [33].

The microenvironment and its biomarkers are not investigated in routine practice. Patients are not discriminated on these criteria, but it is easy to figure that the influence of the microenvironment differs between patients. Furthermore, Rasche et al. hypothesized on an intra-patient heterogeneity [37]; local differences in the microenvironment between focal lesions could select clones with distinct genomic aberrations according to the spatial site. This is another level of heterogeneity which could explain the difference in terms of treatment response.

Since the impact of the immune microenvironment is huge in cancer, myeloma not being an exception, we cannot deny its implications for the prognostic impact and response to treatment. We are not going to speak deeper about this topic that deserves a dedicated review, but we can mention briefly that, for example, an increased Treg cells compartment is associated with worse prognosis [54,55]. It could be interesting to add another degree of prognostic appreciation to the assessment of the relative abundance of immune cells (regulatory versus cytotoxic).

## 4. Conclusions

**Figure 1:**
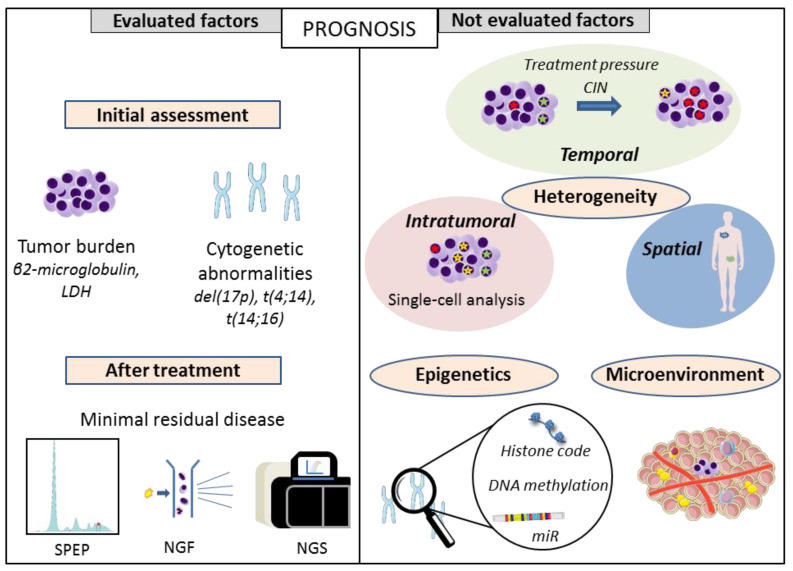
Prognostic factors: routinely evaluated versus not evaluated factors. SPEP: serum protein electrophoresis, NGF: next-generation flow, NGS: next-generation sequencing.

In this review, we described the limits of the current scores that could explain the discrepancy between what is predicted and the outcomes observed in reality (Figure 1). Indeed, the “one-shot” assessment does not take into account the spatial and temporal heterogeneity. Furthermore, MM development is not only influenced by genomic events but also by epigenetic and microenvironmental factors, some of them being described as independent prognostic factors. Nevertheless, clinicians need accessible tools and easy objective criteria for decision making in terms of the therapeutic strategy. Nowadays, these factors are not assessed routinely and there are no guidelines that rely on these elements because it is unclear which biomarkers are those that could really bring discriminant value to current routine assessment. These questions can only be answered by studies that are ancillary to large clinical trials, with the aim of simultaneously dissecting myeloma in all its dimensions and integrating the generated data.
